# Ablation for metastatic lung cancer

**DOI:** 10.1186/1470-7330-15-S1-O29

**Published:** 2015-10-02

**Authors:** Thierry de Baère

**Affiliations:** 1 Imagerie Thérapeutique, Département d'imagerie, Institut de Cancérologie Gustave Roussy, Villejuif, France

## 

Since first report of RFA in lung tumor in year 2000, RFA has been demonstrated to provide 80 to 90% complete ablation for tumors less than 2 cm, with decrease in efficacy for larger tumors. Percutaneous ablation is today a valid option for lung metastases in non surgical candidates with overall survival reported after RFA is in between 56 to 67% at 3 years. Such survival reported is comparable to what reported in large surgical series even if no comparative data exists. Age, disease free interval, tumor size and tumor numbers are independent predictor of survival after RFA of lung metastatses. The same predictive factors have been reported as predictive of survival after surgical metastasectomy.

One of the advanteg of RFA over other technique such as surgery and SBRT is that it can be easily repeated in case of occurrence of new metastases which is difficult with surgery due to the aggressively of the procedure. Subsequent surgical resection are limited by pulmonary reserve. The same applies to stereotaxic radiation therapy where multiple irradiation results in toxicity lo lung parenchyma, skin or mediastinum. Consequently, RFA is today part of routine practice armentarium against lung metastases.

However, many fields remains to be investigate to improve efficacy and to better determine the role fo RFA relative to other therapies. Investigation will be needed :

- To determine the role of various ablation technology such as microwaves, cryotherapy and irreversible electroporation, in comparison to RFA which has been today the most reported technique

- To evaluate the need and benefit from combining local ablation and systemic therapy

- To compare RFA with other local therapy, namely stereotactic body radiation therapy that has today indications very close to percutaneous thermal ablation. These comparisons will help to determine best treatment option in a given situation.

Future trends in treatment of pulmonary metastases will favor minimal aggressive treatments and percutaneous ablation have a role to play. Evidence based medicine supporting the use of lung RFA metastatic disease and tdefining what is the best population to target with ablation or SBRT.

The ideal candidate has less than 3 tumors less than 3 cm.
